# Uses of Next-Generation Sequencing Technologies for the Diagnosis of Primary Immunodeficiencies

**DOI:** 10.3389/fimmu.2017.00847

**Published:** 2017-07-24

**Authors:** Michael Seleman, Rodrigo Hoyos-Bachiloglu, Raif S. Geha, Janet Chou

**Affiliations:** ^1^Division of Immunology, Boston Children’s Hospital, Boston, MA, United States

**Keywords:** primary immunodeficiency, next-generation sequencing, whole exome sequencing, gene panels, genomics

## Abstract

Primary immunodeficiencies (PIDs) are genetic disorders impairing host immunity, leading to life-threatening infections, autoimmunity, and/or malignancies. Genomic technologies have been critical for expediting the discovery of novel genetic defects underlying PIDs, expanding our knowledge of the complex clinical phenotypes associated with PIDs, and in shifting paradigms of PID pathogenesis. Once considered Mendelian, monogenic, and completely penetrant disorders, genomic studies have redefined PIDs as a heterogeneous group of diseases found in the global population that may arise through multigenic defects, non-germline transmission, and with variable penetrance. This review examines the uses of next-generation DNA sequencing (NGS) in the diagnosis of PIDs. While whole genome sequencing identifies variants throughout the genome, whole exome sequencing sequences only the protein-coding regions within a genome, and targeted gene panels sequence only a specific cohort of genes. The advantages and limitations of each sequencing approach are compared. The complexities of variant interpretation and variant validation remain the major challenge in wide-spread implementation of these technologies. Lastly, the roles of NGS in newborn screening and precision therapeutics for individuals with PID are also addressed.

## Introduction

Primary immunodeficiencies (PIDs) are genetic diseases that affect the development and/or function of the immune system, thereby increasing the susceptibility to infectious pathogens, autoimmunity (1), and malignancies (2). Classically, PIDs have been considered monogenic disorders that follow the principles of Mendelian inheritance. However, advances in DNA sequencing technologies have facilitated the identification of multigenic and somatic causes of PIDs and have revealed the wide phenotypic variability of these diseases (3, 4).

The global incidence of PIDs has been estimated to be 1:10,000 live births (5) although this is considered an underestimation due to limited patient access to diagnostic technologies and the challenges of diagnosing patients with atypical clinical presentations (6). Although PIDs are rare diseases from a global perspective, PIDs are more prevalent in areas with highly consanguineous populations due to the predominance of autosomal recessive PIDs. The incidence of consanguineous unions is 65% in the Middle East, significantly higher than what has been found in Europe, the Western Pacific region, and Latin America (5.6, 2.3, and 0.96%, respectively) (7, 8). Correspondingly, the incidence of PID is 20 times greater in Middle Eastern countries compared with North America and Europe (9). Attempts in delineating the epidemiology of PIDs have utilized different data collection methodologies, including clinician registries, hospital/health insurance databases, and population surveys (10–14). These epidemiologic studies have revealed a great need for more clinicians trained in the diagnosis of PIDs as well as access to inexpensive diagnostic technologies, particularly in resource-limited areas of the world (15). To compensate for the uneven distribution of clinical and technical expertise focused on PID, collaborative networks of PID specialists have been established throughout the globe. These networks have facilitated collaborative efforts for identifying novel genetic defects underlying PIDs and improving the diagnosis of PIDs (16).

Traditionally, the initial evaluation of patients with suspected PIDs has consisted of both quantitative and qualitative analysis of the immune system. Laboratories with expertise in the diagnosis of PIDs perform enumeration of lymphocyte subpopulations, lymphocyte proliferation studies, quantification of immunoglobulin levels and vaccine-specific antibody titers, evaluation of complement levels and function, as well as tests for specific pathways (e.g., assays for investigating Toll-like receptor function, neutrophil oxidative burst, or T cell receptor signaling pathways). This approach enables clinicians categorize the phenotype of a patient’s PID, with the aim of prioritizing the most likely causative genetic defects and guiding therapeutic decision (17, 18). However, this approach is time-consuming, costly, and requires viable cells from patients as well as personnel trained in a diversity of laboratory techniques. The shipping of patient blood to tertiary referral centers results in impaired cellular responses and viability that can compromise the accuracy of diagnostic tests (19, 20). Furthermore, the field of PIDs advances rapidly, since mutations in over 200 genes are known to cause PIDs and over 10 novel PIDs are discovered annually (17, 21). Sanger sequencing, which is the conventional approach for gene sequencing, is much slower and more costly than next-generation DNA sequencing (NGS) (22). Therefore, there is a great need for improving patient access to leading-edge diagnostic technologies.

## Impact of NGS on the Genotype–Phenotype Correlation in PIDs

Next-generation DNA sequencing has significantly changed our understanding of PIDs, which are no longer considered purely monogenic diseases following Mendelian patterns of inheritance. By enabling sequencing of the entire exome or genome, NGS has demonstrated the breadth of unusual phenotypes caused by mutations in genes known to cause PID. This is exemplified by patients who have a common variable immunodeficiency (CVID)-like phenotype due to hypomorphic mutations in genes classically associated with severe combined immunodeficiency (SCID). These include *RAG1* (23), *DCLRE1C* (24, 25), and *JAK3* (26). The reported patient with the hypomorphic mutation in *DCLRE1C* did not receive a molecular diagnosis until his second decade of life. There are also reports of hypomorphic mutations in *RAG1* and *RAG2* resulting in a combined immunodeficiency less severe than classical SCID, thus permitting survival into adulthood (27, 28).

The unbiased nature of whole exome sequencing (WES) and whole genome sequencing (WGS) has facilitated the discovery of multigenic PIDs (4). In 28 patients with abnormal degranulation assays, Zhang et al. found heterozygous mutations in two genes associated with familial hemophagocytic lymphohistiocytosis (29). Additionally, the broad spectrum of clinical phenotypes in patients with PIDs may reflect the effects of modifier mutations. As an example, WGS of a patient with LRBA deficiency identified a homozygous mutation in the base excision repair enzyme *NEIL3* (30). Studies of Neil3-deficent mice demonstrated its critical role in maintaining peripheral B cell tolerance and in providing protection against autoimmunity. Synergy of NEIL3 deficiency and LRBA deficiency was proposed as the cause of the patient’s markedly severe clinical phenotype (30).

The high-throughput approach of NGS enables deep sequencing coverage, which refers to the average number of times any given nucleotide is sequenced. This is essential for identifying somatic variants, which occur in only a small subpopulation of cells. Although PIDs are classically considered to be germline defects, somatic mutations have now been recognized as a cause of PIDs (31). For example, autoimmune lymphoproliferative syndrome can arise from somatic mutations in *TNFRSF6, KRAS*, or *NRAS*. Certain mutations in *TNFRSF6*, which encodes the Fas receptor, in the lymphoid lineage have a dominant negative effect that allows increased survival and lymphoproliferation mimicking the phenotype of germline *TNFRSF6* mutations (32, 33). Somatic gain-of-function (GOF) mutations affecting *KRAS* and *NRAS* have been reported to cause RAS-associated leukoproliferative disease, a syndrome that presents with autoimmunity, splenomegaly, and lymphadenopathies, with or without expansion of alpha beta double negative T cells (34, 35). Importantly, some of these somatic mutations have been found only after enriching for specific lymphocyte populations prior to DNA isolation, thus highlighting the importance of cell-specific NGS in some disorders (32).

## NGS Technologies

**Figure 1:**
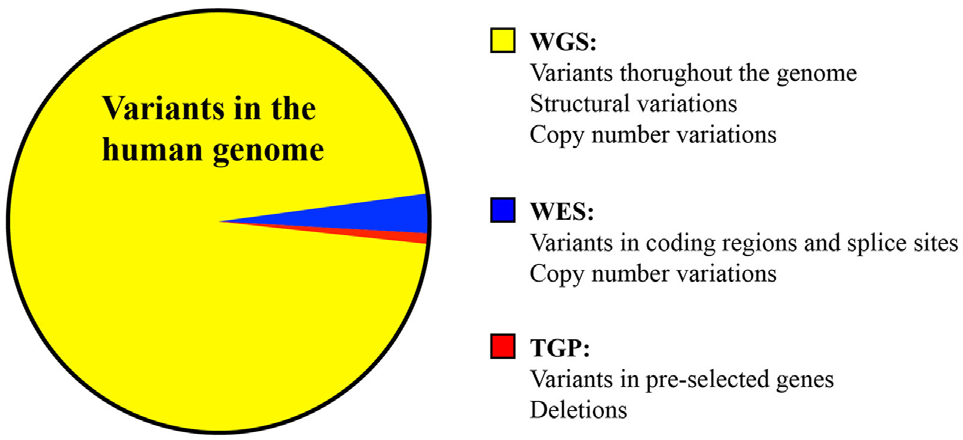
Comparison of the variants identified by whole genome sequencing, whole exome sequencing, and targeted panel sequencing.

Next-generation DNA sequencing has revolutionized the diagnosis of genetic diseases by providing high-throughput and increasingly cost-efficient diagnostic technologies (Figure 1). The most comprehensive NGS technique is WGS, which sequences a patient’s entire genome and enables the identification of variants in exonic and non-coding regions (36). WES is a more focused technology that sequences only the protein-coding regions within a genome, which contain approximately 85% of disease-causing mutations (37). The most focused NGS approach is the targeted gene panel (TGP), which sequences a specific cohort of genes. These three approaches differ primarily in the comprehensiveness of genetic sequencing, which translates into differences in the complexity of data analysis and cost (Table 1). Since the exome contains approximately 30,000 genes, or <2% of the human genome, the exome can be sequenced at a greater depth than the genome at a lower price. The cost of an average exome has been estimated at $800 (38), although the fee for a clinical-grade exome typically exceeds several thousand dollars due to the certifications and regulations applied to clinical testing. A TGP focused on genes specific to a clinical phenotype is even more cost efficient, with studies reporting a range of $250–500 per sample for targeted PID gene panels (39). Barcoding and batching samples in sequencing runs is the key to cost efficiency: increasing the number of samples per sequencing run minimizes the use of NGS reagents, which account for a significant amount of the overall operating costs (22). The decreasing costs of NGS, coupled with the stability of DNA, render NGS a potentially powerful tool even for resource-limited areas.

**Table 1:** Comparison of targeted panels, whole exome sequencing (WES), and whole genome sequencing (WGS).

	Targeted panel	WES	WGS
Target	300 genes	2% of genome	Entire genome
Cost per sample (USD)	$250–500	$800	$1,400–1,600
Variants detected	Variable: depends on the panel size	~20,000	~4,000,000
Advantages	Customizable Lowest cost	Identifies novel genetic causes of primary immunodeficiencies (PIDs) in coding regions Low cost	Identifies novel genetic causes of PIDs in coding and non-coding regions Detects structural variants Most uniform depth of sequencing
Limitations	Variants limited to the pre-selected gene panel Requires updates as new diseases are discovered Cannot detect structural variants	Sequencing depth affected by poor/incomplete exome capture Cannot detect non-coding or structural variants	Highest cost Largest volume of data and the most complex analysis

### Diagnostic Yield

The diagnostic yield of NGS technologies is determined by the limitations specific to each approach. WES and TGP rely on the preparation of libraries containing fragments of patient DNA complementary to the exome or panel of selected genes, respectively (40). Most NGS technologies require PCR amplification of libraries to generate sufficient quantities of DNA for high-throughput sequencing (41). The preparation of incomplete libraries will lead to gaps in sequencing coverage that can potentially miss pathogenic variants. Furthermore, the detection of structural variations, such as large insertions or deletions, translocations, inversions, and copy-number variations, is much more difficult by WES or TGP because the target regions are not contiguous, as they are in WGS (42). The identification of structural variations is important for the diagnosis of PIDs, since pathogenic variations have been commonly identified in large, repetitive genes, such as *DOCK8* and *LRBA* (43).

Studies utilizing patients with PIDs have reported wide variability in the diagnostic yield of NGS approaches. The only study directly comparing WES with WGS in patients with immunodeficiency demonstrated that WGS identified 656 more coding variants than WES in six patient studies; furthermore, WES in this small cohort was not reliable for the detection of copy-number variants, all of which involved non-coding regions (44). WES of patients from 278 families with PIDs achieved a diagnostic yield of 40%, resulting in a modified clinical diagnosis for 50% of patients, and alterations in the therapeutic management for 25% of patients (45). Another study applying WES to 50 patients with CVID found a similar diagnostic yield of 30% (46). The reported diagnostic yields using TGP in patients with PIDs are lower. A TGP of 170 genes associated with PIDs identified a diagnosis in 15% of 26 patients sequenced (47); another study using a TGP of 162 PID genes achieved a diagnostic yield of 25% in 139 patients with PIDs (22). Since TGPs are composed of pre-selected genes, these panels will not identify an unexpected or novel genetic cause of PID, thus leading to a lower diagnostic yield.

### Pitfalls of NGS

Previously published studies utilizing WES or TGPs as diagnostic tools for patients with PIDs show that at least 60% of patients remain undiagnosed (22, 45–47). By comparison, conventional genetic testing, such as Sanger sequencing for single genes, karyotyping, and chromosomal microarrays identifies a diagnosis in only ~15% of patients, thus leaving 85% without a diagnosis (48). Despite the increased diagnostic yield of NGS compared with conventional genetic testing, the fact that the majority of patients lack a diagnosis indicates that deficits in the technologies, data analysis, or our understanding of PIDs remain.

Depending on the depth of sequencing, NGS detects 20,000–50,000 variants per patient sample (49). Sequencing a familial trio or quartet, consisting of the proband and his parents and/or siblings, is a common approach used to narrow the list of candidate mutations (49). However, this approach assumes complete penetrance of the disease. Misidentification of an ostensibly healthy family member as “unaffected” will eliminate all variants in this individual from the candidate variant list, including those pathogenic mutation in the individual that are incompletely penetrant. While incomplete penetrance is well-known to occur in autosomal dominant PIDs, such as CTLA4 haploinsufficiency (50), studies have begun to show variability in the genotype–phenotype association for autosomal recessive diseases as well. For example, a homozygous mutation in *ICOS* resulted in a combined immunodeficiency in the proband, but only mild hypogammaglobulinemia and decreased antibody titers to some but not all vaccines in his sister (51). Both the proband and his sister had absent ICOS expression and severely decreased T follicular helper cells, demonstrating that a deleterious homozygous mutation can translate to clinical variability (51).

Potentially pathogenic variants are prioritized using computational pipelines, comparisons with public databases of polymorphisms, software for predicting the effect of a given variant *in silico* (Polyphen, SIFT, MutationTaster, among others), and knowledge of genetically modified cell lines and animal models (52, 53). Synonymous mutations, which do not alter the amino acid sequence of the protein in production, are typically considered benign variants and eliminated from the candidate mutation list. However, recent studies have shown that synonymous mutations can result in PIDs. A patient with T^−^B^+^NK^low^ SCID was found to have a synonymous mutation in exon 19 of JAK3, which served as a cryptic donor splice site that generated an unstable JAK3 mutant protein (54). In another report, a synonymous mutation in exon 3 of *IL7R* was found to cause aberrant splicing, leading to T^low^B^+^NK^+^ SCID (55). Both of these studies demonstrate that NGS cannot serve as the only diagnostic tool for patients with PIDs, since functional studies are needed to determine the biologic effect of novel mutations.

### Interpretation of Variants

Clinical criteria have been established to standardize the approach for interpreting genetic variants (56). The classification of variants as pathogenic, likely pathogenic, benign, likely benign, or a variant of uncertain significance integrates genetic and biologic criteria. Strong criteria supporting pathogenicity include: a null variant in a gene in which loss of function has been previously shown to cause human disease, studies demonstrating loss of altered protein expression and/or function, previously published evidence of a genotype-disease correlation. Additional criteria include the identification of the variant only in individuals demonstrating the disease phenotype, location of the variant in a highly conserved genomic locus, and *in silico* predictions of pathogenicity. In contrast, benign variants include those with a high (>5%) minor allelic frequency in established databases, those that fail to segregate with the disease phenotype, those with no demonstrated biologic effect through *in vitro* testing. Additionally, mutations in genes expressed strictly in organs without immune function (e.g., genes encoding olfactory receptors) are unlikely to be the cause of PIDs. Variants of uncertain significance are those that do not meet sufficient criteria for classification as pathogenic, likely pathogenic, benign, or likely benign. These variants of uncertain significance include mutations in genes whose relevance to human disease is not yet known. This is particularly relevant to patients with PIDs, since the pace of discovery in the field is rapid: 34 novel genetic causes of PIDs were discovered between 2013 and 2015 (18). GOF variants constitute another challenging category. These mutations are typically predicted to be benign by *in silico* algorithms because these mutations enhance, rather than impair gene function. However, GOF variants in *PIK3CD, STAT1, STAT3*, and *CARD11* result in immune dysfunction or dysregulation, and thus, biologic assays delineating the mechanisms linking a GOF mutation to a disease phenotype are essential (18).

The primary limitation of genomic diagnostics is the lack of functional evidence provided by sequencing alone: functional assays are required to demonstrate the biologic effect of a variant. This is particularly important for non-coding variants whose effects are challenging to predict *in silico*. The importance of functional validation has been underscored in a study of 33 missense mutations in 23 genes essential for immune function. Only 15–20% of those predicted to be deleterious *in silico* were shown to have a pathogenic effect *in vivo* using mouse models (57). Due to the rarity of PIDs, novel defects often occur in single patients and thus lack the burden of proof provided by multiple unrelated patients sharing the same genotype–phenotype correlation. Therefore, criteria have been proposed for establishing the causal relationship between the patient’s genotype and phenotype: firstly, the candidate genotype must not be found in healthy individuals; secondly, the variant must be proven to destroy or significantly impair the expression or function of the gene product; thirdly, the causal relationship between the patient’s genotype and phenotype must be replicated in a relevant cellular or animal model (3). The increasing breadth of public databases, such as the dbSNP, 1,000 genomes, and ExAC databases, enables researchers and clinicians to determine the prevalence of a given mutation in the general population. A diverse array of assays may be required to confirm the biologic effect of mutations identified by NGS. For example, defects impairing gene expression can be evaluated by Western blotting or flow cytometry (58, 59). Receptor activation can be assessed by the phosphorylation of downstream proteins or upregulation of target gene expression (60). Mutations in genes important for lymphocyte activation can be evaluated by assessing upregulation of activation markers, quantifying proliferation in response to mitogens and antigen stimulation, and measure the secretion of cytokines and immunoglobulins after T or B cell activation, respectively (61). Cellular modeling requires the use of patient-derived cells that may not be readily available. The use of induced pluripotent stem cells (iPSCs) is one way to circumvent this obstacle. iPSCs are pluripotent cells derived from terminally differentiated patient cells that can be subsequently re-differentiated into relevant cell types (62–66). As an example, the reprogramming of iPSCs from dermal fibroblasts of TLR3- or UNC-93-deficient patients into neural stem cells, astrocytes, oligodendrocytes, and neurons provided *in vitro* evidence that deficiencies in TLR3 and UNC-93B result in neuronal cell susceptibility to herpes simplex virus 1 (67).

Animal models of PIDs complement cellular studies by enabling investigations of a molecular defect within the context of an *in vivo* immune system on a uniform genetic background. In the field of PIDs, the mouse and zebrafish are the two most commonly used animal models for demonstrating the biologic effect of mutations. Mice are the most frequently used animal model system due to the high level of homology between the mouse and human immune systems, their rapid reproductive rate, and their small size. Mouse models are particularly useful for delineating the contribution of genes with poorly understood contributions to human immunity. This was the case for transferrin receptor 1 (TfR1), a ubiquitously expressed cell surface receptor known to be essential for erythropoiesis (68). A missense mutation impairing TfR1 internalization was shown to result in combined immunodeficiency due to its role in lymphocyte proliferation and class-switching, but permitted normal erythroid development due to the presence of an erythroid cell-specific accessory pathway for TfR1 endocytosis (68). Zebrafish are another *in vivo* system for studying the biologic effect of novel mutations because the majority of human genes have orthologs in the zebrafish genome and their rapid development, high reproductive rate, and transparent bodies are conducive to gene editing and live-imaging studies (69). Zebrafish have been used to model multiple types of SCID using mutants lacking RAG1, ZAP-70, TBX1, JAK3, IL7R, AK2, BCL11B, or EXTL3 (64, 70–72), as well as warts–hypogammaglobulinemia–immunodeficiency–myelokathexis (WHIM) syndrome (73). While these animal models provide invaluable opportunities for delineating the mechanisms driving PIDs *in vivo*, the genetic differences among species constitutes the major limitation of these models. To circumvent this, recent studies have begun to used humanized mice to model PIDs. Humanized mice are generated by transplanting human hematopoietic stem cells (HSCs) into immunodeficient mice, such as the *Il2rg* knockout mice, which then generate human immune cells *de novo*. By combining this approach with iPSC technology, dermal fibroblasts or PBMCs from patients with PIDs can be de-differentiated into iPSCs, which are then re-differentiated into HSCs to generate a humanized mouse model of the patient’s PID. This approach has been used to generate a humanized mouse model of JAK3 deficiency; gene editing using clustered regularly interspaced short palindromic repeat (CRISPR)/CRISPR-associated protein (Cas) successfully corrected the JAK3 mutation and restored T cell development in this model (74). This humanized mouse model therefore provided *in vivo* proof of concept for CRISPR/Cas9 gene editing as a therapeutic strategy for JAK3 deficiency.

**Figure 2:**
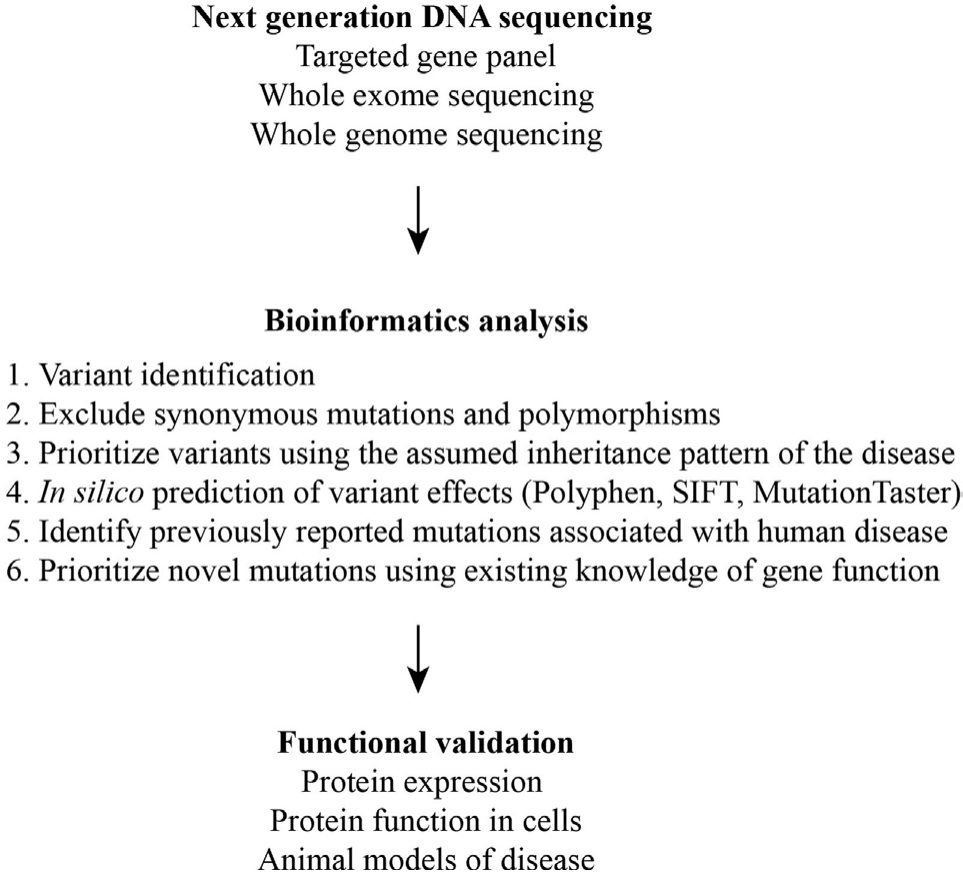
Schematic of the multiple steps required for the identification and validation of disease-causing variants.

Collectively, these studies demonstrate that NGS is only one of many steps in the diagnosis of a PID (Figure 2). DNA, due to its stability, can be easily shipped to diagnostic centers with genomics expertise for NGS and *in silico* interpretation of variants. Therefore, a TGP can serve as a screening test. However, the validation of previously unreported mutations even in well-described genes requires multidisciplinary expertise in molecular and cellular biology, biochemistry, and immunology. Biologic outcomes from a mutation in a poorly characterized or novel gene require cellular and animal modeling of the mutation. This remains a major limitation in the diagnosis of the PIDs in resource-limited areas of the world.

## NGS as a Tool for Precision Medicine in the Field of PIDs

### Newborn Screening (NBS)

The global prevalence and the severity of PIDs indicate a need for improved diagnosis of these disorders, particularly in resource-limited areas of the world. Hematopoietic stem cell transplant (HSCT), the standard cure for patients with SCID, has better outcomes in younger patients. The largest multicenter study of transplantation outcomes in patients with SCID found that the 5-year survival rate and reconstitution were significantly better in patients who received matched sibling donor HSCT, but that the survival rate among infants transplanted prior to 3.5 months of age was high, independent of donor type (75). This has increased the momentum for SCID NBS. The current method of NBS for SCID quantifies T cell receptor excision circles (TRECs), which reflect generation of naïve T cells. Likewise, kappa light chain-deleting recombination excision circles (KRECs) assays are used to measure the generation of naïve B cells. Although relatively inexpensive, these tests do not provide a molecular diagnosis and are limited to defects affecting naïve T and B cell generation. While the current cost of NGS greatly exceeds that of TREC/KREC assays, the falling cost of sequencing may enable targeted panels to serve as an early diagnostic tool, as demonstrated by several feasibility studies. A 2015 study performed NGS on DNA extracted from the dried blood spot to screen for 48 different *CFTR* mutations in 67 newborns with known pathogenic mutations in *CFTR* (76). NGS was in complete concordance with the previously confirmed mutations in this cohort. Another study, in which NGS was used for the screening of inherited metabolic diseases, used a custom gene panel to sequence 97 genes. This panel identified 244 variants in 269 infants, 94% of which were validated by Sanger sequencing (77). Previously undetected pathogenic mutations were also identified in 10 newborns in the same study, suggesting that NGS may increase the sensitivity of NBS.

### Precision Therapeutics

The rapid identification of pathogenic mutation facilitates the use of gene therapy as a potential treatment option, an approach that has been used for ADA-SCID, X-linked SCID, chronic granulomatous disease, and Wiskott–Aldrich Syndrome, with varying degrees of success (78). Additionally, a molecular diagnosis also enables the selection of biologics that target the affected signaling pathway. For example, abatacept, a soluble CTLA4 fusion protein, has been shown to improve autoimmune complications seen in patients with CTLA4 haploinsufficiency or LRBA deficiency, two disorders characterized by immune dysregulation due to impaired expression of the inhibitory molecule CTLA4 (79). Diseases caused by GOF mutations have been shown to be amenable to treatment by targeted inhibitors. Ruxolitinib, an inhibitor of JAK1 and JAK2, improved mucocutaneous candidiasis and autoimmune disease in a patient with a GOF mutations in STAT1 (80). Tocilizumab, an IL-6 inhibitor, improved arthritis and contractures in a patient with an STAT3 GOF mutation, a finding notable because the two patients who were treated with HSCT in this study died of transplant-related complications (81). Patients with GOF mutations in *CXCR4*, resulting in WHIM syndrome, have been treated successfully with the CXCR4 antagonist plerixafor (82). Ongoing clinical trials are underway to assess the efficacy of selective PI3K delta inhibitors in patients with GOF mutations in *PIK3CD*, resulting in activated PI3K delta syndrome (83). Precision therapeutics do not provide germline correction of the molecular defect, but provide an important alternative for patients who have no matched HSC donor, who have a PID with a high rate of HSCT-related mortality, or who have no access to a medical center with expertise in the PID-focused HSCT. The possibility of developing and utilizing targeted biologics depends on the efficient identification of pathogenic mutations in patients with PIDs, thus opening a therapeutic niche for NGS beyond HSCT.

## Conclusion

While NGS has been used extensively in PID-related research, it has a relatively nascent role in clinical immunology. This is due, at least partly, to the time needed for sequencing costs to decrease and to validate the technologies in patients with PIDs. Although the cost of whole genome/exome sequencing remains high for most clinical labs, the field of oncology has shown the effectiveness of targeted NGS panels (84). Targeted NGS allows for a high-throughput, low-cost pipeline with a very short turnaround time due to the limited number of sequenced genes. DNA is inexpensive to isolate and can be easily shipped from remote areas of the world to centers with NGS capabilities, thus circumventing the difficulty inherent in shipping viable cells. The genetic basis of PIDs and the field’s focus on molecular mechanisms, along with the available corrective therapies, render patients with these diseases ideal candidates for NGS. The integration of TGPs into NBS protocols will enable the early diagnosis and treatment of PIDs in a comprehensive manner that is yet achieved by current standard of care.

## Author Contributions

MS, RH-B, RG, and JC wrote and edited the manuscript.

## Conflict of Interest Statement

The authors declare that the research was conducted in the absence of any commercial or financial relationships that could be construed as a potential conflict of interest.
